# Monorchiids (Digenea, Trematoda) of fishes in the Yucatán Peninsula, Mexico, with the description of three new species based on morphological and molecular data[Fn FN1]

**DOI:** 10.1051/parasite/2023015

**Published:** 2023-05-14

**Authors:** Leopoldo Andrade-Gómez, Mirza Patricia Ortega-Olivares, Brenda Solórzano-García, Martín García-Varela, Berenit Mendoza-Garfias, Gerardo Pérez-Ponce de León

**Affiliations:** 1 Departamento de Sistemas y Procesos Naturales, Escuela Nacional de Estudios Superiores Unidad Mérida Km 4.5, Carretera Mérida-Tetiz Ucú Yucatán C.P. 97357 Mexico; 2 Departamento de Zoología, Instituto de Biología, Universidad Nacional Autónoma de México (UNAM), Avenida Universidad 3000, Ciudad Universitaria CP 04510 México City México

**Keywords:** Digenea, SEM, LSU, *cox1*, Haemulidae, Mugilidae

## Abstract

Adult specimens of monorchiids (Digenea) were collected from the intestines of the white grunt, *Haemulon plumierii* Lacepède (Haemulidae), and the white mullet, *Mugil curema* Valenciennes (Mugilidae) from five localities off the Yucatán Peninsula and one locality in the Gulf of Mexico. Some specimens were photographed and sequenced for two molecular markers, the large subunit (LSU) of nuclear rDNA and the cytochrome c oxidase subunit 1 (*cox1*) of mitochondrial DNA. Other specimens were processed for morphological analyses. Newly generated sequences were aligned with other sequences available in GenBank. Bayesian inference and maximum likelihood analyses were implemented using the data sets of LSU and *cox1* independently. Reciprocal monophyly evidenced through phylogenetic analyses, sequence divergence values for both molecular markers, and detailed morphological analyses, including scanning electron microscopy photomicrographs, revealed three new genetic lineages, i.e., species, as parasites of *M. curema*. The three new species are *Sinistroporomonorchis mexicanus* n. sp., *Sinistroporomonorchis yucatanensis* n. sp., and *Sinistroporomonorchis minutus* n. sp. Two additional species of monorchiids were sampled, characterised molecularly, and re-described, namely *Sinistroporomonorchis glebulentus* (Overstreet, 1971) from the white mullet, and *Alloinfundiburictus haemuli* (Overstreet, 1969), from the white grunt.

## Abstract (fr)

Des spécimens adultes de Monorchiidae (Digenea) ont été collectés dans les intestins de la gorette blanche, *Haemulon plumierii* Lacepède (Haemulidae), et du mulet blanc, *Mugil curema* Valenciennes (Mugilidae) de cinq localités au large de la péninsule du Yucatán et d'une localité dans le Golfe du Mexique. Certains spécimens ont été photographiés et séquencés pour deux marqueurs moléculaires, la grande sous-unité (LSU) de l'ADNr nucléaire et la sous-unité 1 de la cytochrome c oxydase (*cox1*) de l'ADN mitochondrial. D'autres spécimens ont été traités pour des analyses morphologiques. Les séquences nouvellement générées ont été alignées avec d'autres séquences disponibles dans GenBank. L'inférence bayésienne et les analyses de vraisemblance maximale ont été mises en œuvre en utilisant les ensembles de données de LSU et *cox1* indépendamment. La monophylie réciproque mise en évidence par des analyses phylogénétiques, des valeurs de divergence de séquence pour les deux marqueurs moléculaires et des analyses morphologiques détaillées, y compris des photomicrographies au microscope électronique à balayage, a révélé trois nouvelles lignées génétiques, c'est-à-dire des espèces, qui sont parasites de *M. curema*. Les trois nouvelles espèces sont *Sinistroporomonorchis mexicanus* n. sp., *Sinistroporomonorchis yucatanensis* n. sp. et *Sinistroporomonorchis minutus* n. sp. (Monorchiidae). Deux espèces supplémentaires de Monorchiidae ont été échantillonnées, caractérisées moléculairement et redécrites, à savoir *Sinistroporomonorchis glebulentus* (Overstreet, 1971) du mulet blanc et *Alloinfundiburictus haemuli* (Overstreet, 1969) de la gorette blanche.

## Introduction

Members of Monorchiidae Odhner, 1911 represent a diverse group of trematodes that infect the gastro-intestinal tract mostly of marine fish, with approximately 265 valid species allocated to six subfamilies [30]. Monorchiids are morphologically characterised by complex terminal genitalia armed with recurved spines, vitelline follicles in restricted fields, and well-developed uterine coils [11]. The genetic library for species allocated to the family has increased significantly in the recent years; several new species have been described following an integrative taxonomy approach, most of them from Australian marine fish [18, 25–27, 29–31]. In a recent study, Wee *et al.* [28] performed one of the most comprehensive studies for species in the genus *Lasiotocus* Looss 1907, a taxonomically problematic group within the Monorchiidae. In their study, four new genera were erected in order to accommodate the 34 species previously recognised as members of *Lasiotocus*.

In Mexico, monorchiids of marine fish have been scarcely studied. To date, only five species have been reported, parasitizing species of the families Haemulidae, Carangidae, Balistidae, or Serranidae, specifically *Diplomonorchis alexanderi* (Arai, 1963) Bijukumar, 1997; *D. caballeroi* (Zhukov, 1983); *Pseudohurleytrema longitestis* (Bravo-Hollis, 1956) Yamaguti, 1971; *Ametrodaptes mexicanus* Bravo-Hollis, 1956, and *Monorchicestrahelmins bupharynx* (Bravo-Hollis, 1956). During this investigation of the parasite fauna of marine and estuarine fish across the Yucatán Peninsula, specimens of monorchiids were collected and processed for morphological and molecular analyses. We describe three new species of monorchiids and report for the first time *S. glebulentus* as a parasite of mugilids in the Yucatán Peninsula*.* Additionally, we generated sequences for the first time for species in the genus *Alloinfundiburictus* Wee, Cutmore, Pérez-del-Olmo & Cribb, 2020. Both species are redescribed.

## Materials and methods

### Ethical standards

Specimens were collected under the Cartilla Nacional de Colector Científico (FAUT 0202) issued by the Secretaría del Medio Ambiente y Recursos Naturales (SEMARNAT), to MGV. Fish were humanely euthanised following the protocols described in the 2020 edition of the American Veterinary Medical Association (AVMA) Guidelines for the Euthanasia of Animals.

### Sample collection

Monorchiids were collected from the intestines of 45 individuals of *M. curema*, 16 individuals of *Mugil* sp., and 17 individuals of *H. plumierii* distributed in five localities of the Yucatán Peninsula, plus one locality in the Gulf of Mexico (Table 1). Fishes were collected in January 2020 and May 2022 using cast nets, kept alive and examined for helminths a few hours after capture. Individual fish were euthanised by spinal severance (pithing), following the procedures described by the American Veterinary Medical Association [3], necropsied, and their organs immediately examined under a stereomicroscope. Monorchiids were recovered from the intestines, fixed in hot distilled water, and preserved in 100% ethanol for morphological and molecular analyses.

**Table 1:** Monorchiids spp. recorded in this study with localities, hosts and GenBank accession number.


	Locality	Georeference	Host	Hosts infected/Hosts examined	Species of monorchiids	LSU	*cox1*
1	Alvarado, Veracruz	18° 46′ 47.82′′ N, 95° 44′ 50.1′′ W	*Mugil* sp.	3/8	*Sinistroporomonorchis mexicanus* n. sp	OQ672296	
			*Mugil* sp.	3/8	*Sinistroporomonorchis minutus* n. sp.	OQ672305	OQ674208
2	Nuevo Campechito, Campeche	18° 38′ 55.8′′ N, 92° 28′ 2.5′′ W	*Mugil curema*	5/7	*Sinistroporomonorchis mexicanus* n. sp.	OQ672289*–*95	OQ674202–04
3	Laguna Términos, in Isla Aguada, Campeche	18° 45′ 26.6′′ N, 91° 30′ 54.8′′ W	*Mugil curema*	3/4	*Sinistroporomonorchis glebulentus*	OQ672284*–*86	OQ674199–201
			*Mugil curema*	2/4	*Sinistroporomonorchis minutus* n. sp.	OQ672306*–*08	OQ674209
4	Champotón, Campeche	19° 21′ 40.3′′ N, 90° 43′ 5.37′′ W	*Mugil curema*	8/11	*Sinistroporomonorchis yucatanensis* n. sp.	OQ672297*–*302	OQ674205
5	Celestún, Yucatán	20° 50′ 53.5′′ N, 90° 24′ 22′′ W	*Mugil curema*	3/5	*Sinistroporomonorchis yucatanensis* n. sp.	OQ672303*–*04	OQ674206–07
			*Mugil curema*	3/5	*Sinistroporomonorchis minutus* n. sp.	OQ672309*–*10	
6	Off the coast of Sisal, Yucatan	21° 08′ 1.5′′ N, 90° 07′ 55.9′′ W	*Haemulon plumierii*	4/17	*Alloinfundiburictus haemuli*	OQ672280*–*83	OQ674197*–*98
			*Mugil curema*	3/9	*Sinistroporomonorchis glebulentus*	OQ672287*–*88	

### Morphological analyses

Some unflatten specimens were post-fixed in hot formalin to harden the tegument. Specimens were dehydrated through graded alcohol series, stained with Mayer’s paracarmine (Merck, Darmstadt, Germany), cleared with methyl salicylate, and mounted on microscope slides with Canada balsam. Mounted specimens were examined under a bright field Leica DM 1000 LED microscope (Leica, Wetzlar, Germany), and drawings were made using a drawing tube attached to the microscope. Measurements were taken using Leica Application Suite microscope software (Leica) and are given in micrometres (μm). Measurements are shown in Table 2, except for *Alloinfundiburictus haemuli* (Overstreet, 1969) Wee, Cutmore, Pérez-del-Olmo & Cribb, 2020 whose measurements are shown within the re-description. Holotype, paratypes and voucher specimens were deposited in the Colección Nacional de Helmintos (CNHE), Instituto de Biología, Universidad Nacional Autónoma de México, Mexico City.

**Table 2:** Comparative metrical data for *Sinistroporomonorchis* spp. Measurements expressed as the range followed by the mean in parentheses.


	*Sinistropormonorchis glebulentus*	*Sinistropormonorchis glebulentus*	*Sinistropormonorchis lizae*	*Sinistroporomonorchis mexicanus* n. sp.	*Sinistroporomonorchis yucatanensis* n. sp.	*Sinistroporomonorchis minutus* n. sp.

Reference	[16]	This study	[8]^1^, [2]^2^	This study	This study	This study
Type-Locality	Mississippi, USA	Isla Aguada, Campeche	Xiamen, China	Nuevo Campechito, Campeche	Celestún, Yucatán	Celestún, Yucatán
Type-Host	*Mugil cephalus*	*Mugil curema*	*Liza carinata*	*Mugil curema*	*Mugil curema*	*Mugil curema*
*n*	28	2 vouchers; 2 *photogenophores*	20 (13^1^, 7^2^)	8	6	7

BL	458–1,124 (725*)	921–1140 (990) (n=4)	528–732	678–795 (731)	600–858 (763)	325–408 (360)
BW	201–310 (229*)	240–280 (261)	248–327	127–227 (166)	130–172 (157)	114–155 (129)
BL/BW	3.16*	3.2–4.3 (3.86)	2.03*^1^	3.5–5.8 (4.6)	3.6–5.55 (4.91)	2.6–3 (2.8)
FO	119–275 (199*)	210–259 (235)	178*^1^	166–210 (180)	202–245 (223)	102–123 (112)
FO/BL^%^	21–34 (25*)	22.6–26.7 (23.7)	30*^1^	22.9–26.4 (20)	24.5–33.6 (29.5)	25–34.4 (31.3)
HI	186*	597–789 (666)	321*^1^	439–519 (490)	320–553 (461)	172–236 (193)
HI/BL^%^	25.7*	63.4–69.2 (67.2)	55*^1^	64.5–69.5 (67)	53.3–65.3 (60)	52–57.8 (53.6)
OSL	35–79 (67*)	67–80 (76)	56–89	51–69 (60)	56–63 (58)	38–45 (42)
OSW	58–102 (73*)	72–86 (79)	70–104	64–81 (70)	65–81 (74)	48–70 (54)
VSL	60–102 (76*)	83–101 (93)	74–116	52–73 (61)	71–84 (77)	45–66 (54)
VSW	67–107 (85*)	84–106 (98)	82–116	56–74 (65)	56–85 (72)	51–72 (60)
OSL/VSL	0.88*	0.79–0.86 (0.82)	0.83*^1^	0.83–1.19 (0.99)	0.66–0.81 (0.76)	0.68–0.84 (0.78)
OSW/VSW	0.85*	0.75–0.95 (0.81)	0.83*^1^	0.91–1.27 (1.08)	0.9–1.16 (1.05)	0.78–1.11 (0.93)
PL	19–56 (19*)	13–31 (22)	0–7	10–20 (15)	18–28 (23)	0–13 (9)
PHL	26–40 (39*)	32–43 (37)	22–46	25–45 (32)	32–42 (37)	23–32 (27)
PHW	30–51 (45*)	45–51 (48)	32–50	38–48 (42)	47–55 (50)	34–44 (37)
PHL/OSL	0.59*	0.4–0.55 (0.48)	0.48*^1^	0.47–0.65 (0.53)	0.57–0.69 (0.64)	0.56–0.71 (0.64)
PHW/OSW	0.58*	0.56–0.68 (0.61)	0.41*^1^	0.55–0.64 (0.59)	0.62–0.84 (0.68)	0.61–0.75 (0.69)
OEL	23–63 (42*)	73–88 (79)	16–36	36–65 (45)	52–144 (89)	25–48 (33)
OEL/BL^%^	5.9*	6.6–9.5 (8.1)	7*^1^	4.7–8.7 (6.2)	6.9–20 (12)	7.4–11.7 (9.2)
CANT	168*	203–229 (216)	136*^1^	136–170 (150)	174–261 (206)	99–124 (108)
CANT/B^%^	23.2*	20–24 (21.9) (n=4)	23.3*^1^	18.4–22.8 (21)	22.3–37 (27.4)	28–34.6 (30.1)
CEND	229*	215–277 (246) (n=2)	256*^1^	208–356 (312)	23–73 (47)	41–62 (51)
CEND/B^%^	6–50 (31.6*)	22.4–30 (26.3)	43.7*^1^	30.6–47.7 (42.5)	2.8–8.5 (6.1)	10.7–17.1 (14.2)
TL	123–233 (174*)	222–261 (242)	110–210	101–161 (126)	98–147 (116)	57–72 (65)
TW	47–119 (113*)	120–126 (123)	92–154	67–90 (78)	67–85 (74)	45–63 (53)
TVSU	82*	106–205 (156)	2*^1^	72–144 (95)	152–253 (202)	38–62 (45)
TVSU/B^%^	11.3*	11–22.2 (16.7)	0.4*^1^	9.6–18.9 (13.1)	21.2–31.8 (26.4)	9.5–17.8 (12.6)
TANT	346*	416–503 (460)	265*^1^	295–384 (333)	424–553 (504)	194–231 (212)
TANT/B^%^	47.6*	43.4–54.6 (49)	42*^1^	39.5–50.5 (45.7)	61–72.8 (66.3)	50.7–66.1 (59)
TEND	211*	265–283 (274)	85–244	250–314 (285)	76–207 (154)	59–128 (85)
TEND/B^%^	14–43 (29*)	28.7–29.5 (29.2)	97.5*^1^	36.7–41.8 (39.1)	12.6–24.4 (19.7)	17–31.3 (23.2)
CSL	180–393 (263*)	302–322 (312)	100–250	187–292 (228)	183–236 (204)	100–147 (129)
CSW	37–68 (42*)	50–62 (55) (n=3)	34–58	36–44 (40)	46–66 (58)	30–35 (33)
CSL/BL^%^	22–38 (36*)	32.7–33.6 (33.2)	25*^1^	25–36.7 (31.2)	24.4–30 (27)	28.7–41.7 (36)
SVL	58*	77–82 (80)	63*^1^	33–101 (63)	48–63 (57)	27–40 (33)
SVW	36*	36–46 (41)	48*^1^	24–61 (38)	30–48 (38)	21–27 (24)
SVL/CSL^%^	22*	23.9–27.1 (25.5)	43.3*^1^	17.6–36.1 (27)	25.1–34.4 (28)	19.7–36 (25.8)
PPL	76*	32–38 (35)	78*^1^	16–41 (26)	32–63 (46)	23–42 (29)
CL	159*	170–199 (185)	53*^1^	125–150 (140)	96–110 (101)	50–79 (65)
CW	21*	25–27 (26)	19*^1^	20–27 (23)	33–46 (38)	14–28 (20)
CL/CSL^%^	45–68 (60*)	56.2–61.8 (59)	36.6*	51.3–70.5 (61.9)	46.6–52.9 (49.5)	41.3–56.2 (50.2)
TOL	88–135 (116*)	152–156 (154)	38–100	91–150 (118)	93–127 (111)	57–90 (69)
TOW	37–72 (45*)	57–67 (62)	20–35	37–53 (46)	41–58 (50)	30–36 (33)
ATOL	48*	81–100 (91)	34*^1^	52–96 (69)	46–79 (62)	29–52 (38)
ATOW	27*	31–32 (32)	22*^1^	27–48 (36)	29–48 (39)	26–33 (28)
PTOL	48*	54–55 (55)	31*^1^	39–60 (52)	42–71 (56)	29–52 (40)
PTOW	36*	47–61 (54)	14*^1^	29–46 (37)	32–54 (44)	23–36 (29)
OL	44–170 (137*)	151–158 (155)	108–162	53–146 (107)	65–84 (74)	25–64 (48)
OW	28–109 (76*)	63–71 (67)	48–100	38–90 (59)	43–48 (45)	17–44 (34)
OANT	284*	352–373 (363)	173*^1^	220–283 (247)	359–524 (442)	163–207 (188)
OANT/BL^%^	39.2*	36.7–40.4 (38.6)	29.5*^1^	30–38.6 (33.8)	54.2–62.6 (58.7)	43.6–59.6 (52)
OEND	211*	421–457 (439)	270*^1^	349–409 (381)	167–310 (240)	110–176 (129)
OEND/BL^%^	29.1*	45.7–47.7 (46.7)	46.2*^1^	47.6–55.4 (52.2)	27.8–36.6 (31.5)	31.5–43.1 (35.7)
VR	76*	117–133 (123)	48–123	84–131 (97.3)	90–129 (111)	40–69 (57)
VR/BL^%^	10.5*	10.3–14.4 (12.3)	19.1*^1^	11.2–17.2 (13.3)	12.7–16.2 (14.5)	11.4–21.2 (15.9)
EL	16–26	14–23 (21)	18–22	13–23 (18)	19–38 (30)	11–33 (24)
EW	9–12	7–11 (9)	8–11	7–12 (10)	7–12 (9)	6–15 (9)

^*^Estimated from the published drawing. BL body length, BW maximum body width, FO forebody, HI hindbody, OSL oral sucker length, OSW oral sucker width, VSL ventral sucker length, VSW ventral sucker width, PL prepharynx length, PHL pharynx length, PHW pharynx width, OEL oesophagus length, CANT pre-bifurcal zone, CEND postcaecal field length, TL testis length, TW testis width, TVSU testis to ventral sucker, TANT pre-testicular zone, TEND Post-testicular zone, CSL cirrus sac length, CSW cirrus sac width, SVL seminal vesicle length, SVW seminal vesicle width, PPL pars prostatic length, CL cirrus length, CW cirrus width, TOL terminal organ length, TOW terminal organ width, ATOL anterior terminal organ length, ATOW anterior terminal organ width, PTOL posterior terminal organ length, PTOW posterior terminal organ width, OL ovary length, OW ovary width, OANT pre-ovarian zone, OEND post-ovarian zone, VL vitellarium range, EL egg length, EW egg width, ^% ^represented in percentage, ^1,2^Estimated from publication [8] or [2].

For scanning electron microscopy (SEM), specimens were dehydrated in a graded ethanol series, critical point dried, sputter coated with gold, and examined with a Hitachi Stereoscan Model SU1510 scanning electron microscope operating at 10 kV at the Laboratorio de Microscopia y Fotografía de la Biodiversidad, Instituto de Biología, Universidad Nacional Autónoma de México.

### Amplification and sequencing of DNA

**Figure 1:**
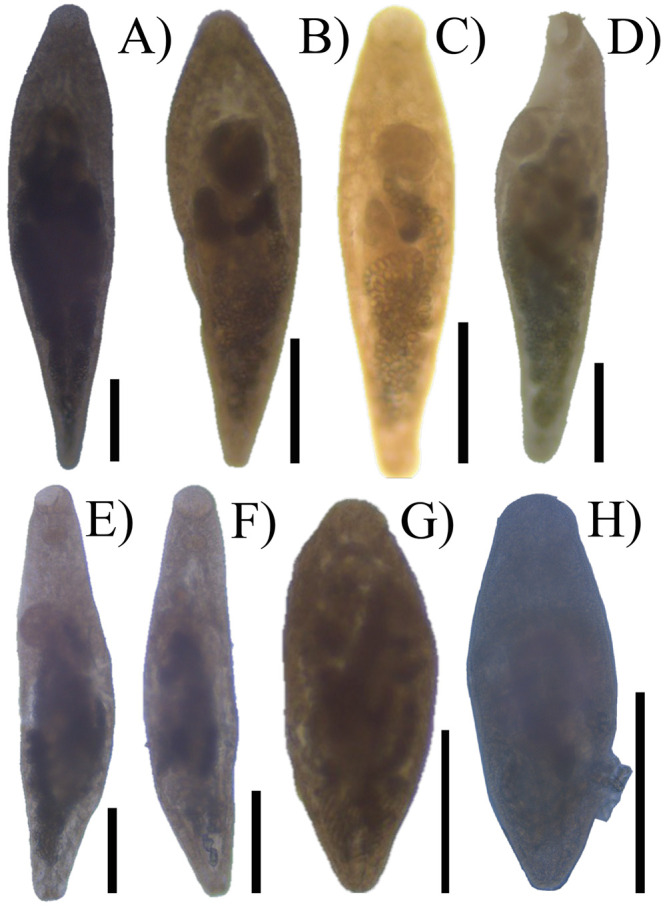
*Photogenophores* of *Sinistroporomonorchis* spp.: *S glebulentus* (**A** OQ672284/OQ674199, **B** OQ672285/OQ674200); *S. mexicanus* n. sp. (**C** OQ672296, **D** OQ672294); *S*. *yucatanensis* n. sp. (**E** OQ672297, **F** OQ672298/OQ674205); *S*. *minutus* n. sp. (**G** OQ672307, **H** OQ672310). Scale bars, 200 µm. In parentheses, GenBank accession numbers (see Table 1 for host and localities).

Prior to DNA extraction, each specimen was mounted on a semi-permanent slide to obtain a reference image under a bright field Leica DM 1000 LED microscope (Leica, Wetzlar, Germany). Each image was linked with its genomic DNA, a *photogenophore* *sensu* Andrade-Gómez and García-Varela [1] (Fig. 1). Specimens were then removed from the slide and placed individually in tubes with a digestion solution for DNA extraction at 56 °C overnight. The digestion solution contained 10 mM Tris-HCl (pH 7.6), 20 mM NaCl, 100 mM Na_2_ EDTA (pH 8.0), 1% sarkosyl, and 0.1 mg/mL proteinase K. Following digestion, DNA was extracted from the supernatant using DNAzol reagent (Molecular Research Center, Cincinnati, OH, USA), according to the manufacturer’s instructions. For amplification, the D1–D3 domains of LSU from rDNA were amplified using forward 391 5′–AGCGGAGGAAAAGAAACTAA–3′) and reverse 536 (5′–CAGCTATCCTGAGGGAAAC–3′) primers [5]. Additionally, a fragment of *cox1* was amplified using primers designed by McNamara *et al*. [12], *cox1*tremF (5′–TTCACKTTGGATCATAAGCGT–3′) and Mon.mt3 (5′–ACCATAAACATRTGRTG–3′). PCRs (25 µL) consisted of 1 µL of each primer (10 µM), 2.5 µL of 10× PCR Rxn buffer, 1.5 µL of 2 mM MgCl_2_, 0.5 µL of dNTPs (10 mM), 16. 375 µL of water, 2 µL of genomic DNA and 1 U of Taq DNA polymerase (Platinum Taq, Invitrogen Corporation, São Paulo, Brazil). PCR cycling parameters included denaturation at 94 °C for 1 min, followed by 35 cycles at 94 °C for 1 min, annealing at 50 °C for LSU and at 48 °C for *cox1* for 1 min, and extension at 72 °C for 1 min, followed by postamplification incubation at 72 °C for 10 min. Sequencing reactions were performed using the initial primers plus two internal primers, 504 (5′–CGTCTTGAAACACGGACTAAGG–3′), 503 (5′–CCTTGGTCCGTGTTTCAAGACG–3′) [22], and ABI Big Dye (Applied Biosystems, Boston, MA, USA) terminator sequencing chemistry, and reaction products were separated and detected using an ABI 3730 capillary DNA sequencer. Contigs were assembled and base-calling differences resolved using CodonCode Aligner version 9.0.1 (CodonCode Corporation, Dedham, MA, USA).

### Alignments and phylogenetic analyses

LSU rDNA and *cox1* mtDNA sequences obtained in the current study were independently aligned with data from another Monorchiidae species downloaded from the GenBank dataset, plus six species from three families, i.e., Haploporidae, Deropristidae, and Lissorchiidae used as outgroups for rooting the trees (see Supplementary Table 1). The alignment consisted of 81 sequences with 1292 nucleotides for the LSU rDNA, and 55 sequences with 588 nucleotides for *cox1.* Alignments were constructed using Clustal W software [24] with default parameters and adjusted manually with Mesquite software [10].

The phylogenetic analyses were performed using Maximum Likelihood (ML) and Bayesian Inference (BI) methods. The ML was carried out with RAxML version 7.0.4 [21] and Bayesian Inference (BI) analyses were inferred with MrBayes version 3.2.7 [6] using the online interface: Cyberinfrastructure for Phylogenetic Research (CIPRES) Science Gateway v3.3 [13]. The best model was estimated with the Akaike information criterion (AIC) using the jModel Test version 0.1.1 program [19], which predicted the best model for the LSU dataset to be GTR + I + G, and for the *cox1* dataset to be GTR + G. To test nodal ML support, each node was run with 1,000 bootstrap replicates. BI analyses included two simultaneous runs of Markov Chain Monte Carlo (MCMC) for 10 million generations, sampling every 1,000 generations, with a heating parameter value of 0.2 and a “burn-in” of 25%. Trees were drawn using FigTree program v.1.3.1 [20]. The genetic divergence among taxa was estimated using uncorrected “p” distances with MEGA v. 6. [23] (see Supplementary Table 2).

## Results

### Morphological descriptions

*Alloinfundiburictus* Wee, Cutmore, Pérez-del-Olmo & Cribb, 2020

#### *Alloinfundiburictus haemuli* (Overstreet, 1969) Wee, Cutmore, Pérez-del-Olmo & Cribb, 2020 (Fig. 2)

**Figure 2:**
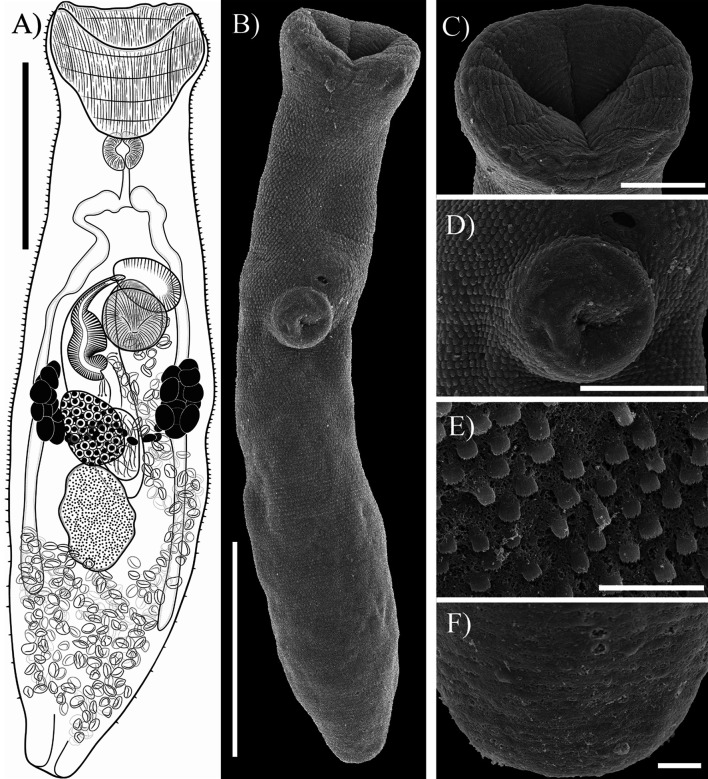
*Alloinfundiburictus haemuli* (Overstreet, 1969) Wee, Cutmore, Pérez-del-Olmo & Cribb, 2020 from *Haemulon plumierii* (**A**) Whole worm voucher; Scanning electron micrographs of voucher; (**B**) whole worm; (**C**) Oral sucker; (**D**) Ventral sucker; (**E**) Tegumental spines; (**F**) Posterior region. Scale bars (μm) = (A, B) 200; (C, D) 50; (E, F) 10.

Type host: *Haemulon plumierii* Lacepède, white grunt, Haemulidae

Additional host: *Haemulon sciurus* Shaw, Bluestriped grunt; *Brachygenis chrysargyreum* (Günther), Smallmouth grunt; *H. flavolineatum* Desmarest, French grunt, Haemulidae

Site of infection: Intestine

Type locality: Biscayne Bay, Florida, USA

Additional localities: Off the coast of Sisal, Yucatán (this study), Puerto Morelos Reef National Park, Quintana Roo, Mexico (as *Lasiotocus haemuli*) [9]

Voucher material: CNHE 11837

Representative DNA sequences: OQ672280 *–* OQ672283 (LSU), OQ674197 *–* OQ674198 (*cox1*).

Re-description (Based on seven mature individuals. Range followed by the mean in parentheses): Body elongated, 725−1008 (813) long, 163−225 (204) wide, 3.22−5.14 (4.01) times longer than wide, usually with a constriction posterior to oral sucker level. Tegument thin, armed with small serrate spines, decreasing in posterior body end. Forebody relatively short 227−360 (298), 31.3−44.2% (36.7%) of body length. Oral sucker terminal, funnel-shaped, 114−159 (142) long, 109−226 (166) wide. Ventral sucker small, round, slightly anterior to middle of body, 58−82 (67) long, 57−76 (65) wide. Prepharynx not visible in most specimens, one individual with prepharynx 22 long. Pharynx muscular, small, spherical, overlapping slightly with oral sucker, 34−44 (40) long, 35−49 (43) wide, 25−30% (29%) of oral sucker length, 17−40% (27%) of oral sucker width. Oesophagus shorter than pharynx 34−48 (42), in three specimens slightly longer than pharynx, occupies 4.1−6% (5.2%) of body length. Intestinal bifurcation in forebody, well anterior to ventral sucker; pre-bifurcal zone 188−236 (212) occupies 23.4−28.2% (26.1%) of body length. Intestinal caeca long, terminate in post-testicular zone 178−299 (225), or 22.2−33.6% (27.7%) of body length, from posterior end of body.

Testis single, irregular, in middle of hindbody, 101−157 (134) long, 81−125 (95) wide, separated by 7.1−16.4% (11.9%) of body length from ventral sucker, 57−137 (97); pre-testicular zone 398−556 (460), or 50.2−64.4% (56.7%) of body length; post-testicular zone 180−324 (242), or 24.4−34.4% (29.6%) of body length. Cirrus-sac slightly arcuate, in middle of body, mostly intercaecal, posterior end slightly overlaps ovary and testes extending around dextral of ventral sucker, 125−304 (245) long, 50−81 (69) wide, representing 17−41.9% (30.3%) of body length. Seminal vesicle ovoid, unipartite, 50−117 (89) long, 38−61 (50) wide, occupies 29.9−47.2% (37.1%) of cirrus-sac length. Pars prostatica simple, with prostatic cells, 37−58 (49) long. Cirrus (eversible ejaculatory duct) relatively thick, armed with robust spines 51−126 (101) long, 24−48 (39) wide, occupies 37.2−43.4% (41.3%) of cirrus-sac length. Genital atrium short, simple and unspined. Genital pore small, median or just sinistral or dextral to midline, immediately anterior to ventral sucker.

Ovary smooth, ellipsoidal to slightly triangular, in midbody, posterior to ventral sucker, slightly dextral to median line, partially overlaps testis, 73−100 (87) long, 58−76 (66) wide; pre-ovarian zone 317−499 (403), or 40−56.9% (49.8%) of body length; post-ovarian zone 248−440 (340), or 34.2−50% (41.6%) of body length. Canalicular seminal receptacle not observed. Vitellarium composed of two masses of follicles in lateral fields, between ventral sucker and ovary of body, 72−117 (97) long, or 9−16.1% (12.2%) of body length; follicles round or irregularly shaped, densely clustered. Uterus mostly restricted to hindbody extending past caecal termination, thin-walled, extensive, with coils mostly indiscernible; ascending coil forms metraterm, enters terminal organ at posterior end. Terminal organ sinistro-ventral to cirrus-sac, bipartite, 88−127 (106) long, 63−89 (71) wide, comprising unspined posterior chamber, and spined anterior section. Posterior chamber spherical dorsal to ventral sucker, containing fibrous mass, 59−85 (73) long, 41−58 (52) wide. Anterior section armed with long and thin spines, in middle unspined; 49−78 (67) long, 51−83 (65) wide. Eggs operculate, 13−23 (17) long, 7−11 (9) wide. Excretory vesicle tubular shaped. Excretory pore terminal.

Remarks: Our specimens collected from *Haemulon plumierii* in Yucatán were identified as *Alloinfundiburictus haemuli* by having features that are consistent with the diagnosis of the original description [15]. Our specimens possess a funnel-shaped oral sucker, and most traits are morphologically very similar to those of the original description. Additionally, specimens possess an unlobed ovary, which is a diagnostic trait of the genus *Alloinfundiburictus*, as described by Wee *et al*. [28].

In addition, the specimens sampled in the present study were obtained from the type-host and the same overall biogeographical region (Gulf of Mexico) as the original report. This parasite has also been reported from three species of haemulids from the Puerto Morelos Reef National Park in the Caribbean Sea [9]; however, for samples in that report the vitellarium distribution seems to be asymmetrical. This trait is characteristic of *A. asymmetricus* (Fischthal, 1977), a species described from *H. flavolineatum* off the coast of Belize, also in the Caribbean Sea. The species reported by López-Zacarías *et al*. [9] was collected from the same biogeographical region and the same host species as *A. asymmetricus*. Additionally, DNA sequences are necessary to corroborate the specific status of these specimens.

The taxonomy of monorchiids has been shown to be rather complex. The genus *Lasiotocus* is a clear example (see [28]). According to Paschoal *et al*. [17] at least 11 species of *Lasiotocus* are found in haemulids (although some species have also been reported from other host species), nine from the Gulf of Mexico, Caribbean Sea, and southwestern Atlantic, and only two from the eastern Pacific coast. Two of them, i.e., *Lasiotocus lintoni* (Manter, 1931) and *L. minutus* (Manter, 1931) were retained in the genus *Lasiotocus*, whereas the remaining nine species were transferred to the genus *Infundiburictus* Wee, Cutmore, Pérez-del-Olmo & Cribb, 2020 by Wee *et al*. [28]. Thus, in the western Atlantic at least eight species of *Infundiburictus* can be found parasitizing about 16 species of haemulids, 10 of the genus *Haemulon*, four of the genus *Anisotremus* Gill, and two of the genus *Orthopristis* Girard. Clearly a detailed molecular phylogenetic analysis is needed to disentangle the species diversity within the genus, and to address aspects of host specificity.

*Sinistroporomonorchis* Wee, Cutmore, Pérez-del-Olmo & Cribb, 2020

## *Sinistroporomonorchis glebulentus* (Overstreet, 1971) Wee, Cutmore, Pérez-del-Olmo & Cribb, 2020 (Fig. 3)

**Figure 3:**
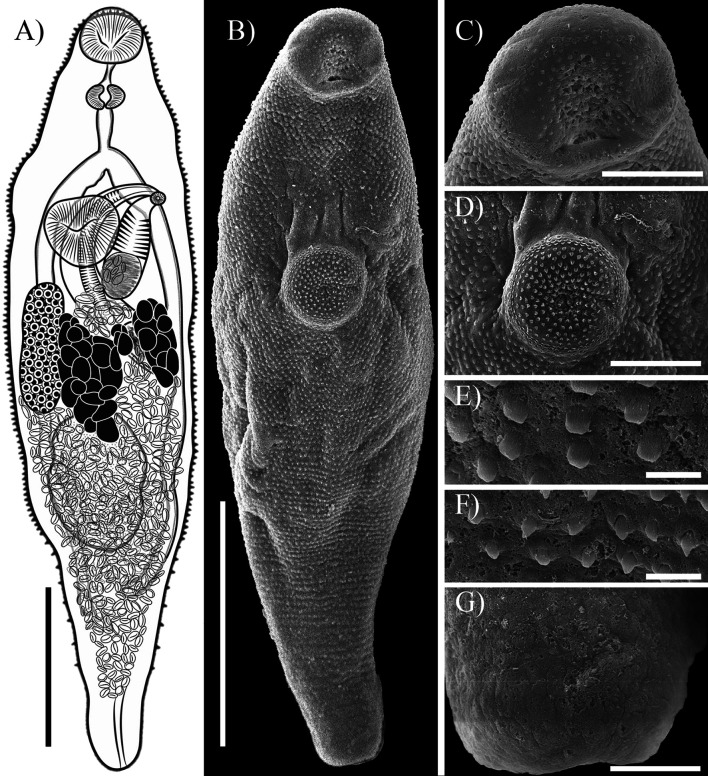
*Sinistroporomonorchis glebulentus* (Overstreet, 1971) Wee, Cutmore, Pérez-del-Olmo & Cribb, 2020 from *Mugil curema* (**A**) Whole worm voucher; Scanning electron micrographs of voucher; (**B**) Whole worm; (**C**) Oral sucker; (**D**) Ventral sucker; (**E**) Tegumental spines at middle of body; (**F**) Tegumental spines at third of body; (**G**) Posterior region. Scale bars (μm) = (A, B) 200; (C) 40; (D) 50; (E, F) 5; (G) 20.

Type host: *Mugil cephalus* Linnaeus, grey mullet, Mugilidae

Additional host: *Mugil curema* Valenciennes, white mullet, Mugilidae

Site of infection: Intestine

Type locality: Mississippi Sound, Mississippi, USA

Additional localities: Dauphin Island, Alabama, USA; Laguna Términos, in Isla Aguada, Campeche; Off the coast of Sisal, Yucatán in Mexico

Voucher material: CNHE 11838

Representative DNA sequences: OQ672284 *–* OQ672288 (LSU), OQ674199 *–* OQ674201 (*cox1*)

Re-description (Based on two vouchers and two *photogenophores*. Measurements in Table 2.): Body elongated to fusiform; 3.86 times longer than wide. Tegument thin, armed with small spines decreasing in number towards posterior body end of body; blunt spines at level of ventral sucker; pointed spines in hindbody (Figs. 3E–3F). Forebody short, occupies 23.7% of body length. Oral sucker round, with ventral aperture. Ventral sucker round, slightly anterior to middle of body. Prepharynx present. Pharynx muscular, spherical, 48.2% of oral sucker length, 61% of oral sucker width. Oesophagus moderately short and straight, occupies 8.1% of body length. Intestinal bifurcation near level of ventral sucker; pre-bifurcal zone occupies 21.9% of body length. Intestinal caeca long, terminate in hindbody at testicular or post-testicular level, 26.3% of body length from posterior body end. Testis single, elongate, in hindbody, 16.7% of body length from ventral sucker, overlaps one or both caeca; pre-testicular zone 49.0% of body length; post-testicular zone 29.2% of body length. Cirrus-sac arcuate, dorsal to ventral sucker extending to middle body, slightly overlaps one or both caeca, representing 33.2% of body length. Seminal vesicle ovoid, unipartite; occupies 25.5% of cirrus-sac length. Pars prostatica simple, with few prostatic cells observed. Cirrus (eversible ejaculatory duct) relatively thick, armed with robust spines with thorn-shaped, occupies 59% of cirrus-sac length. Genital atrium muscular, unspined, simple. Common genital pore small, sinistral to midline, near of ventral sucker. Ovary smooth, elongate, posterior to level of ventral sucker, dextral, somewhat separated from testis, partially overlaps right caecum ventrally; pre-ovarian zone 38.6% of body length; post-ovarian zone 46.7% of body length. Canalicular seminal receptacle not observed. Vitellarium composed of two lateral fields of large, round, densely clustered follicles, distributed at level of ovary and testis, posterior to level of ventral sucker extending to level of testis, occupies 12.3% of body length. Uterus mostly restricted to hindbody, thin-walled, extensive, ventral to ovary, testis, caeca and part of cirrus-sac, with coils mostly indiscernible; ascending coil forms metraterm and enters terminal organ at posterior end. Terminal organ sinistro-ventral to cirrus-sac, bipartite, comprising unspined posterior chamber, and spined anterior section. Posterior chamber spherical sinistral and posterior to ventral sucker, containing fibrous mass. Anterior section armed with long and thin spines, and in middle unspined. Eggs lightly tanned, operculate, unfilamented. Excretory vesicle tubular, concretions observed in only one specimen (Fig. 1A). Excretory pore terminal.

Remarks: Our specimens from *Mugil curema* from Isla Aguada, Campeche were identified as *Sinistroporomonorchis glebulentus* in possessing features that are consistent with the diagnosis of the original description, including the size and shape of the body and the cirrus-sac, the presence of a terminal organ located sinistrally to the cirrus-sac, an ovary located dextrally in middle-body, and the position and size of testis (see Table 2; [16]). Additionally, our specimens were collected from a mullet in the same genus as the type host (from *M. curema* instead of *M. cephalus*), in the same biogeographical region (Gulf of Mexico).

## *Sinistroporomonorchis mexicanus* n. sp. (Fig. 4)

**Figure 4:**
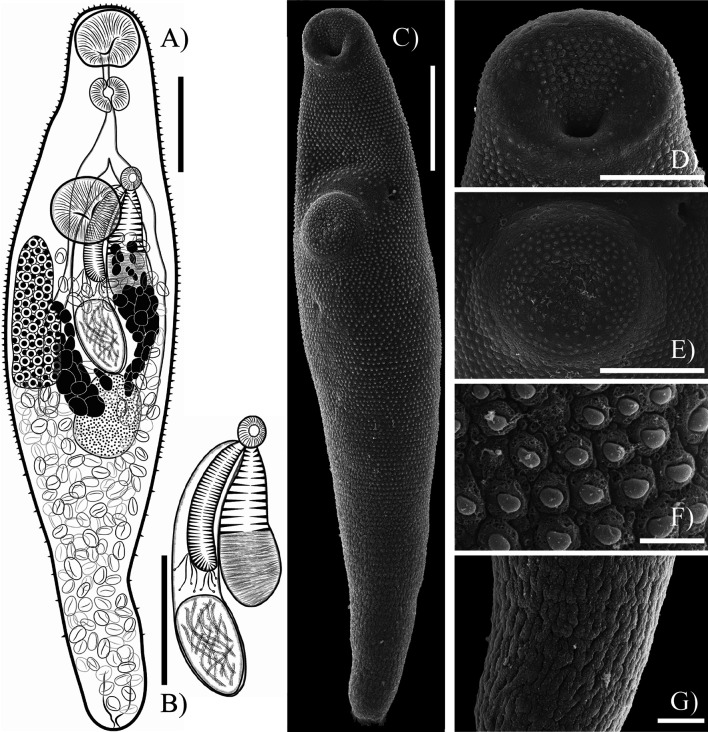
*Sinistroporomonorchis mexicanus* n. sp. from *Mugil curema* (**A**) Whole worm holotype, ventral view; (**B**) terminal genitalia of holotype; Scanning electron micrographs of voucher; (**C**) Whole worm; (**D**) Oral sucker; (**E**) Ventral sucker; (**F**) Tegumental spines; (G) Posterior region. Scale bars (μm) = (A, B, C) 100; (D, E) 40; (F) 5 μm; (G) 15.

urn:lsid:zoobank.org.act:04AE64E6-0C8A-47AB-99D2-4F5D980029B2

Type host: *Mugil curema* Valenciennes, white mullet, Mugilidae

Additional host: *Mugil* sp.

Site of infection: Intestine

Type locality: Nuevo Campechito, Campeche (18° 38′ 55.8″ N, 92° 28′ 2.5″ W)

Additional locality: Alvarado, Veracruz, Mexico

Specimens deposited: 1 Holotype (CNHE 11839); 7 paratypes (CNHE 11840)

Etymology: The specific epithet *mexicanus* refers to the country where the species was found for the first time.

Representative DNA sequences: OQ672289 *–* OQ672296 (LSU), OQ674202–OQ674204 (*cox1*)

Description (Based on eight mature individuals. Measurements in Table 2): Body elongated, slightly fusiform, pointed at posterior end, widest at midbody, 4.58 times longer than wide. Tegument thin, armed with small blunt spines decreasing in number posteriorly. Forebody relatively short, occupies 24.6% of body length. Oral sucker round, with ventral aperture. Ventral sucker roughly rounded, located at first third of body. Prepharynx present. Pharynx muscular, spherical, 53.2% of oral sucker length, 59.7% of oral sucker width. Oesophagus moderately short, occupies 6.2% of body length. Intestinal bifurcation in forebody, well anterior to level of ventral sucker; pre-bifurcal zone occupies 20.6% of body length. Intestinal caeca, long, terminate at level of testis, slightly posterior to midbody, 42.5% of body length from posterior end of body. Testis single, subspherical, slightly posterior to midbody, 18.9% of body length from ventral sucker, overlaps one caeca; pre-testicular zone occupies 45.7% of body length; post-testicular zone occupies 39.1% of body length. Cirrus-sac arcuate, dorsal to ventral sucker, extending to anterior of margin of testis, mostly intercaecal, slightly overlaps one caeca, representing 31.2% of body length. Seminal vesicle ellipsoidal, unipartite, occupies 27% of cirrus-sac length. Pars prostatica simple, with few prostatic cells observed; prostatic cells only observed to unite with pars prostatica towards anterior end. Cirrus (eversible ejaculatory duct) relatively thick, armed with robust spines, occupies 62% of cirrus-sac length. Genital atrium unspined. Common genital pore small, sinistral to midline, anterior to ventral sucker. Ovary smooth, elongate, slightly irregular, in anterior half of hindbody, typically distinctly posterior to ventral sucker, partially overlaps right caecum ventrally; pre-ovarian zone 33.8% of body length; post-ovarian zone 52.2% of body length. Canalicular seminal receptacle not observed. Vitellarium composed of two, never confluent, lateral fields of large, round or irregularly shaped, densely clustered follicles, distributed from level of mid-ovary to level of mid-testis, dorsal to ovary, occupies 13.3% of body length. Uterus mostly restricted to hindbody, thin-walled, extensive, ventral to ovary, testis, caeca and part of cirrus-sac, with coils mostly indiscernible; ascending coil forms metraterm and enters terminal organ at posterior end. Terminal organ sinistro-ventral to cirrus-sac, bipartite, comprising unspined posterior chamber, and spined anterior section. Posterior chamber spherical sinistral and posterior to ventral sucker, containing fibrous mass. Anterior section armed with long and thin spines, and in middle is unspined. Eggs lightly tanned, operculate, unfilamented. Excretory vesicle Y-shaped. Excretory pore terminal.

### *Sinistroporomonorchis yucatanensis* n. sp. (Fig. 5)

**Figure 5:**
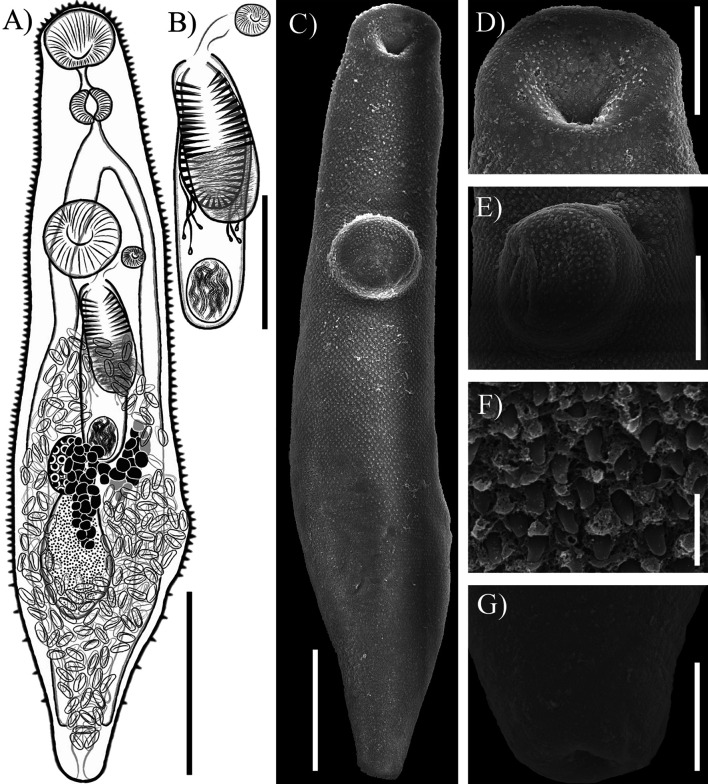
*Sinistroporomonorchis yucatanensis* n. sp. from *Mugil curema* (**A**) Whole worm holotype, ventral view; (**B**) terminal genitalia of holotype; Scanning electron micrographs of voucher; (**C**) Whole worm; (**D**) Oral sucker; (**E**) Ventral sucker; (**F**) Tegumental spines; (**G**) Posterior region. Scale bars (μm) = (A) 200; (B, C) 100; (D) 40; (E) 50; (F) 5; (G) 30.

urn:lsid:zoobank.org.act:04E281A6-3C45-4279-B010-1CFF392C6A7A

Type host: *Mugil curema* Valenciennes, white mullet, Mugilidae

Site of infection: Intestine

Type locality: Celestún, Yucatán (20° 50′ 53.5″ N, 9° 24′ 22″ W)

Additional locality: Champotón, Campeche, Mexico

Specimens deposited: 1 Holotype (CNHE 11841); 5 paratypes (CNHE 11842)

Etymology: The specific epithet refers to the overall area where the species was found, the Yucatán Peninsula.

Representative DNA sequences: OQ672297 –OQ672304 (LSU), OQ674205–OQ674207 (*cox1*)

Description (Based on six mature individuals. Measurements in Table 2.): Body elongated, tapered at posterior end, widest in hindbody; 4.9 times longer than wide. Tegument thin, armed with small, pointed-spines decreasing in posterior end of body. Forebody relatively short, occupies 29.5% of body length. Oral sucker round, with ventral aperture. Ventral sucker roughly round, located at first third of body. Prepharynx present. Pharynx muscular, subspherical, 63.7% of oral sucker length, 68% of oral sucker width. Oesophagus moderately short, mostly straight, occupies 12% of body length. Intestinal bifurcation in mid- to posterior forebody, well anterior to ventral sucker; pre-bifurcal zone occupies 27.4% of body length. Intestinal caeca very long, terminate in posterior end of body, 6.1% of body length from posterior end of body. Testis single, large, entire, slightly ellipsoidal, slightly posterior to middle of hindbody, 26.4% of body length from ventral sucker, overlaps one caeca; pre-testicular zone occupies 66.3% of body length; post-testicular zone occupies 19.7% of body length. Cirrus-sac subcylindrical, in middle of body, typically mostly intercaecal, posterior end slightly overlaps to vitellarium, representing 27% of body length. Seminal vesicle spherical, unipartite, occupies 28% of cirrus-sac length. Pars prostatica simple, with few prostatic cells observed; prostatic cells only observed to unite with pars prostatica towards anterior end. Cirrus (eversible ejaculatory duct) relatively thick, subcylindrical, armed with robust spines, occupies 49.5% of cirrus-sac length. Genital atrium unspined, simple. Common genital pore small, sinistral to midline, at anterior or posterior ventral sucker level. Ovary smooth, subspherical, in middle of hindbody, typically distinctly posterior to ventral sucker, slightly anterodextral to and partially overlaps testis; pre-ovarian zone occupies 58.7% of body length; post-ovarian zone occupies 31.5% of body length. Canalicular seminal receptacle not observed. Vitellarium composed of two, never confluent, asymmetrical lateral fields of large, round shaped, densely clustered follicles, distributed from slightly anterior of ovary level to middle of testis level, ventral to ovary and posterior end of cirrus-sac, occupies 14.5% of body length. Uterus restricted to hindbody, thin-walled, extensive, ventral to ovary, testis, caeca and part of cirrus-sac, with coils mostly indiscernible; ascending coil forms metraterm and enters terminal organ at posterior end. Terminal organ ventral to cirrus-sac, bipartite, comprising unspined posterior chamber, and spined anterior section. Posterior chamber subspherical at middle of body, containing fibrous mass. Anterior section armed with long and thin spines like cirrus-sac spines, and in middle unspined. Eggs lightly tanned, operculate, unfilamented. Excretory vesicle Y-shaped, containing concretions in some specimens (Fig. 1F), typically intercaecal. Excretory pore terminal.

### Sinistroporomonorchis minutus n. sp. (Fig. 6)

**Figure 6:**
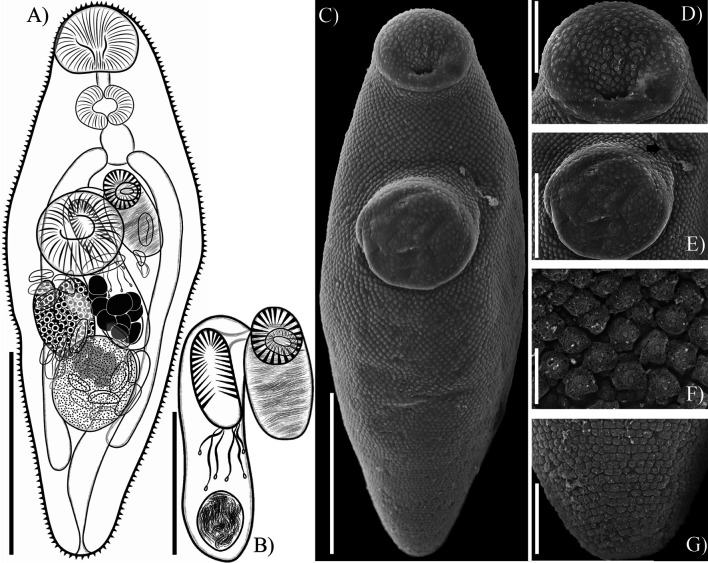
*Sinistroporomonorchis minutus* n. sp. from *Mugil curema* (**A**) Whole worm holotype, ventral view; (**B**) terminal genitalia of holotype; Scanning electron micrographs of voucher; (**C**) Whole worm; (**D**) Oral sucker; (**E**) Ventral sucker; (**F**) Tegumental spines; (**G**) Posterior region. Scale bars (μm) = (A) 100; (B) 50; (C) 100; (D) 30; (E) 50; (F) 5; (G) 20. Arrow marks genital pore.

https://zoobank.org/urn:lsid:zoobank.org:act:45102548-B11D-497A-86B6-8580CB3AD836

Type host: *Mugil curema* Valenciennes, white mullet, Mugilidae

Additional host: *Mugil* sp.

Site of infection: Intestine

Type locality: Celestún, Yucatán (20° 50′ 53.5″ N, 90° 24′ 22″ W)

Additional two localities: Alvarado, Veracruz; Laguna de Términos, in Isla Aguada, Campeche, Mexico

Specimens deposited: 1 Holotype (CNHE 11843); 6 paratypes (CNHE 11844)

Etymology: The specific epithet is derived from the Latin word *minutus* and refers to the small size of the body.

Representative DNA sequences: OQ672305 OQ672305–OQ672310 (LSU), OQ674208–OQ674209 (*cox1*)

Description (Based on 7 mature individuals. Measurements in Table 2.): Body fusiform, widest in middle of body, 2.77 times longer than wide. Tegument thin, armed with small spines throughout body. Forebody relatively short, occupies 31.3% of body length. Oral sucker round, with ventral aperture. Ventral sucker round located slightly anterior to the half of the body. Prepharynx short, only absent in one specimen. Pharynx muscular, slightly ovoid, 64% of oral sucker length, 69.3% of oral sucker width. Oesophagus short, straight, occupies 9.2% of body length. Intestinal bifurcation well anterior to ventral sucker; pre-bifurcal zone occupies 30.1% of body length. Intestinal caeca, long, terminate in post-testicular zone, in posterior of body, 14.2% of body length from posterior end of body. Testis single, large, entire, subspherical, in middle of hindbody, 12.6% of body length from ventral sucker, overlaps one or both caeca; pre-testicular zone occupies 59% of body length; post-testicular zone occupies 23.2% of body length. Cirrus-sac slightly arcuate subcylindrical, dorsal to ventral sucker extending to anterior of testis level, typically mostly intercaecal, slightly overlaps one or both caeca, occupying 36% of body length. Seminal vesicle subspherical, unipartite, occupies 25.8% of cirrus-sac length. Pars prostatica simple, with few prostatic cells observed; prostatic cells only observed to unite with pars prostatica towards anterior end. Cirrus (eversible ejaculatory duct) relatively thick, subcylindrical, armed with robust spines, occupies 50.2% of cirrus-sac length. Genital atrium unspined and simple. Common genital pore small, sinistral to midline, immediately anterior to ventral sucker. Ovary somewhat bipartite and subspherical, in anterior half of hindbody, immediately posterior to ventral sucker, partially overlaps or just posterior to ventral sucker in some specimens, anterodextral to and partially overlaps testis, partially overlaps right caecum ventrally; pre-ovarian zone occupies 52% of body length; post-ovarian zone occupies 35.7% of body length. Canalicular seminal receptacle not observed. Vitellarium composed of two fields in tandem of large round or irregularly shaped, densely clustered follicles, distributed at level of ovary and testis, never extends posteriorly beyond testis, one field dorsal to testis, occupies 15.9% of body length. Uterus mostly restricted to hindbody, thin-walled, extensive, ventral to ovary, testis, caeca and part of cirrus-sac, with coils indiscernible; ascending coil forms metraterm and enters terminal organ at posterior end. Terminal organ sinistro-ventral to cirrus-sac, bipartite, comprising unspined posterior chamber, and spined anterior section. Posterior chamber slightly elongate at middle of body, sinistral to ventral sucker, containing fibrous mass. Anterior section armed with long and robust spines, and in middle unspined. Eggs few, lightly tanned, operculate, unfilamented. Excretory vesicle tubular. Excretory pore terminal.

### Remarks on *Sinistroporomonorchis*

Prior to this study, only two species of *Sinistroporomonorchis* were recognised [28, 32], both from mugilids distributed in America and Asia, *S. glebulentus* (Overstreet 1971) and *S. lizae* (Liu, 2002), respectively. *Sinistroporomonorchis glebulentus* (type-species) was described from *Mugil cephalus* (L.) in estuarine waters of the Gulf of Mexico, whereas *S. lizae* was described from *Planiliza carinata* Valenciennes, in the Taiwan Strait, China. With the inclusion of the three new species described herein, the genus *Sinistroporomonorchis* now contains five species with consistent morphological features as described by Wee *et al*. [28]. These authors reported a distinctly sinistral genital pore, an unspecialised oral sucker, a short oesophagus, and restricted vitelline masses as the diagnostic traits of the genus.

The three new species described in this study, although sampled from the same geographical area in the Yucatán Peninsula, and from the same host (*Mugil curema*), can be easily differentiated by the following characters. *Sinistroporomonorchis* *mexicanus* n. sp. differs from *S. yucatanensis* n. sp. and *S. minutus* n. sp. by having a larger cirrus (125−150 *vs* 96−110 and 50−79, respectively); a larger post-caecal field (208−356 *vs* 23−73 and 41−62, respectively); a larger post-testicular zone (250−314 *vs* 76−207 and 59−128, respectively); and a larger post-ovarian zone (349−409 *vs* 167−310 and 110−176, respectively). Furthermore, *S. minutus* can be easily distinguished from *S. yucatanensis* n. sp. by the small size of the body and by having smaller internal organs. For instance, in *S. minutus* n. sp. the body length is 325−408, whereas in *S. yucatanensis* n. sp. the body is 600−858. Accordingly, *S. minutus* n. sp. exhibits a smaller forebody, hindbody, oral sucker, ventral sucker, and prepharynx (Table 2).

The three newly described species can be readily distinguished from *S. lizae* (originally described as *Lasiotocus lizae* and recently transferred to *Sinistroporomonorchis* by Wee *et al*. [28]) by possessing an oval body instead of an elongate to fusiform body shape. The body width in *S. lizae* is wider than the three newly described species (248−327 *vs* 127−227, 130−172 and 114−155, for *S. mexicanus* n. sp.*, S. yucatanensis* n. sp., and *S. minutus* n. sp., respectively). In *S. lizae*, the body is 2.03 times larger than wide, whereas in the three new species the body is 2.6−5.8 times larger, 3.6−5.55, 2.6−3, respectively. For *S. lizae*, the ventral sucker is larger than in the three new species (74−116 length *vs* 52−73, 71−84, 45−66). Finally, *S. lizae* is the only species distributed in Asia, while the three new species described herein are found in the western Atlantic coast across coastal areas of the Yucatán Peninsula (Table 2).

*Sinistroporomonorchis glebulentus* was originally described in the genus *Lasiotocus* by Overstreet [16], as a parasite of *Mugil cephalus* off Mississippi and Alabama, USA in the Gulf of Mexico. Recently, it was transferred to *Sinistroporomonorchis* and designated as the type-species by Wee *et al*. [28]. Overstreet [16] characterised *S*. *glebulentus* as a species possessing conspicuous concretions in the excretory vesicle. Nonetheless, Liu [8] reported the presence of these concretions in *S. lizae*, which are also observed concretions in *S. yucatanensis* n. sp. (see Fig. 1F). Therefore, the diagnostic character of *S*. *glebulentus* appears to be unreliable for differentiation among congeneric species. However, our specimens identified as *S. glebulentus* can be readily distinguished from the other four species by having an overall larger body: 921−1140 in *S. glebulentus* *vs* 528−732, 678−795, 600−858 and 325−408 in *S. lizae*, *S. mexicanus* n. sp., *S. yucatanensis* n. sp. and *S. minutus* n. sp., respectively. Moreover, some internal organs are also larger in *S. glebulentus* than in the other four species, such as hindbody, testis, cirrus-sac and terminal organ (Table 2).

### Molecular data and phylogenetic analysis

#### LSU

**Figure 7:**
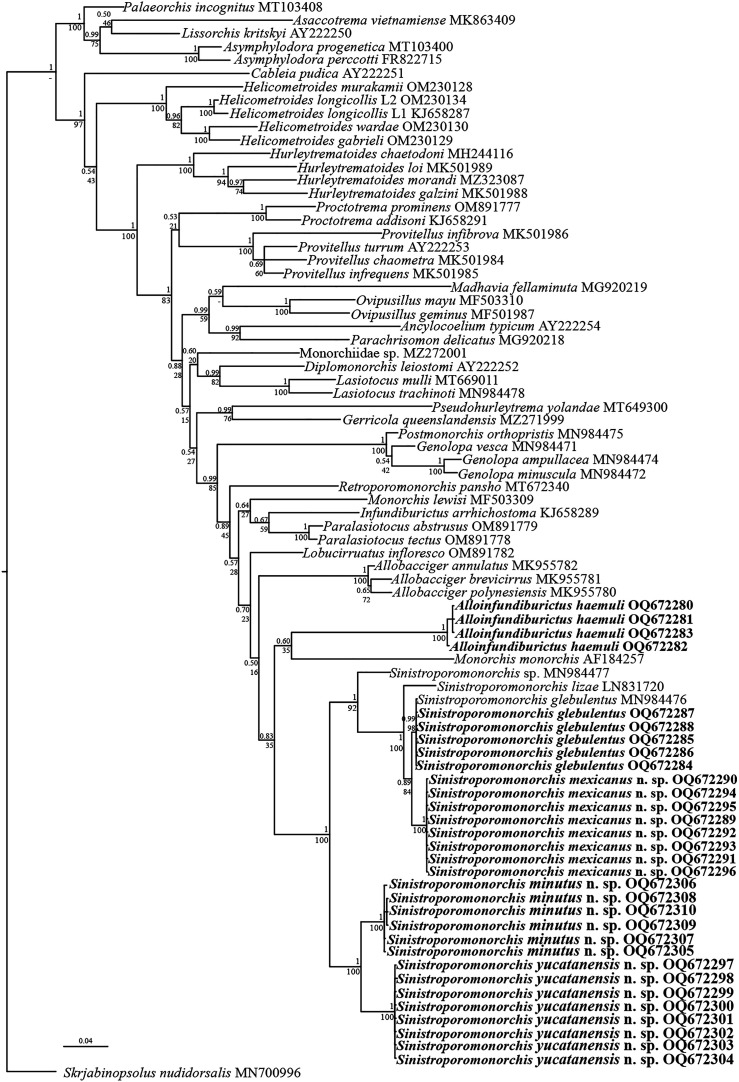
Consensus Bayesian Inference and Maximum Likelihood trees inferred with LSU data set; numbers near internal nodes show posterior probabilities (BI) and ML bootstrap clade frequencies.

The LSU data set included 81 sequences and comprised 1,292 nucleotides. The alignment (trimmed to the shortest sequence) included six sequences of species of Deropristidae and Lissorchiidae used as outgroups. The phylogenetic analyses inferred with ML and BI recovered similar topologies (Fig. 7). The analyses show that Monorchiidae is monophyletic; and *Alloinfundiburictus haemuli* is resolved as the sister group of *Monorchis monorchis* (Stossich, 1890) Looss, 1902 albeit with low nodal support (0.60/35). These two species are yielded as the sister group to species of *Sinistroporomonorchis*, although the clade is moderately to poorly supported (0.83/35). The genus *Sinistroporomonorchis* is monophyletic, strongly supported (1/100), and contains two major clades. The first clade is well-supported (1/92) and includes four lineages corresponding to *S. glebulentus, S. mexicanus* n. sp., *S. lizae*, and *Sinistroporomonorchis* sp. The five newly generated sequences of *S. glebulentus* nested with the sequence of *S. glebulentus* (GenBank MN984476), a parasite of *Mugil curema* from North Carolina, USA (see [18]), demonstrating conspecificity. This species appeared as the sister group of *S. mexicanus* n. sp. (8 isolates). Furthermore, these two species were recovered as the sister group of *S. lizae* (GenBank LN831720) and the three of them as the sister group of ‘*Lasiotocus* sp.’ ex *Menidia menidia* (GenBank MN984477) from New Jersey, USA [18]. Based on the position of this species on the tree and the fact that relationships among these taxa are highly supported, herein we recognised it as a member of *Sinistroporomonorchis* sp. The second clade includes two of the new species, i.e., *S. yucatanensis* n. sp. (8 isolates) and *S. minutus* n. sp. (6 isolates), with strong nodal support (1/100).

The genetic divergence estimated for LSU between *S. glebulentus* and *S. mexicanus* n. sp. ranged from 0.9 to 1%, whereas the divergence between these two species and their sister taxa (*S. lizae* and *Sinistroporomonorchis* sp.) varied from 4.2 to 5% (Supplementary Table 2). The genetic divergence estimated for LSU between *S. yucatanensis* and *S. minutus* ranged from 2.6 to 2.9%. The intraspecific genetic divergence among isolates of the new species we described in this study was 0%, 0.08%, and 0.1%, for *S. mexicanus*n. sp., *S. yucatanensis*n. sp. and *S. minutus*n. sp., respectively. The interspecific genetic divergence among the three new species varied from 2.6 to 6.9%.

#### cox1

**Figure 8:**
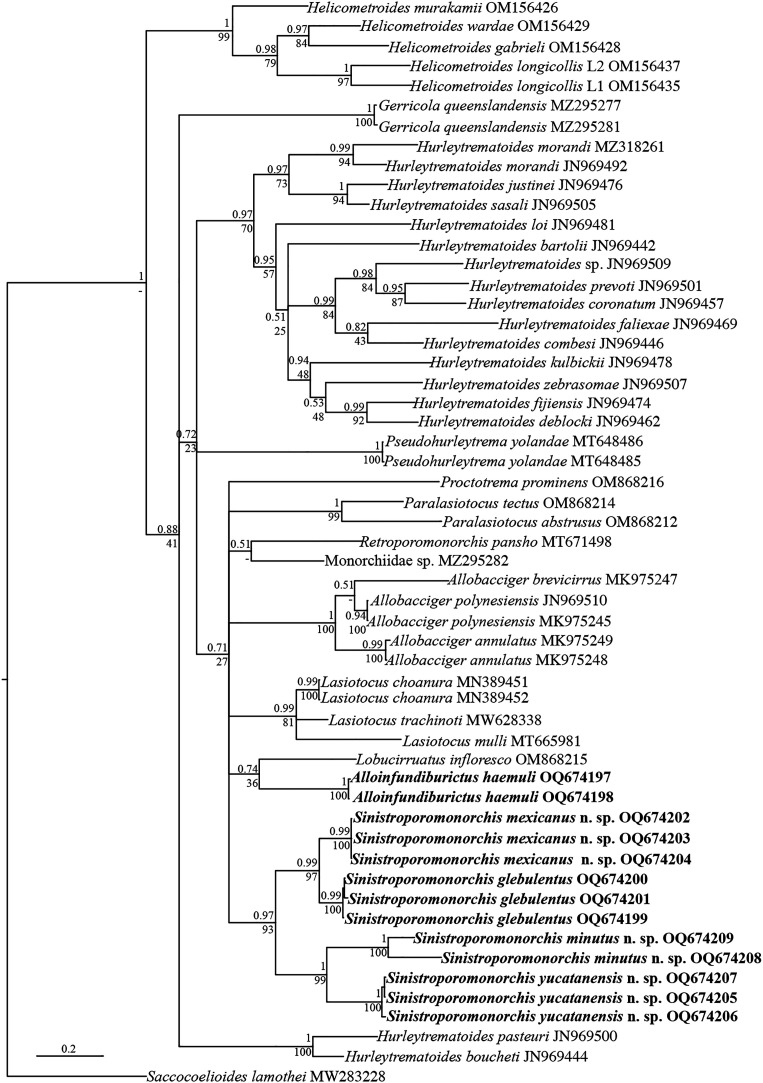
Consensus Bayesian Inference and Maximum Likelihood trees inferred with *cox1* data set; numbers near internal nodes show posterior probabilities (BI) and ML bootstrap clade frequencies.

The *cox1* data set included 55 individuals and comprised 588 nucleotides. The alignment (trimmed to the shortest sequence) only included one sequence of Haploporidae that was used as the outgroup. The phylogenetic analyses of *cox1* inferred with ML and BI recovered similar topologies (Fig. 8). The analyses also showed that Monorchiidae is monophyletic. The 11 new sequences of specimens of *Sinistroporomonorchis* were recovered as a monophyletic assemblage, with strong nodal support (0.97/93). Like in LSU analyses, *Sinistroporomonorchis* was divided in two major clades, also well-supported. The first major clade included three sequences of *S. glebulentus* as the sister group to three sequences of *S. mexicanus* n. sp. The second major clade included the isolates of the other two new species, *S. yucatanensis* n. sp. and *S. minutus* n. sp.

The genetic divergence estimated for *cox1* between the two species that conformed the first major clade (*S. glebulentus* and *S. mexicanus* n. sp.) varied from 9 to 9.4%, whereas the divergence within the second major clade reached 14.4 to 15% between *S. yucatanensis* n. sp. and *S. minutus* n. sp. Finally, the genetic variation between the two major clades was high, ranging from 14.4 to 18.2%.

## Discussion

Members of Monorchiidae are typical components of the helminth fauna of marine fishes and are distributed worldwide. The family now comprises some 268 species, of which 115 species are parasites of four families of marine fishes, namely Carangidae, Chaetodontidae, Haemulidae and Mullidae, representing 42% of all monorchiid species [11, 32]. In contrast, only seven species, including the three newly described species, have been recorded in mugilids in localities of China, Brazil, USA, and Mexico, representing just 2.6% of monorchiid diversity across the globe [7, 28].

The phylogenetic analyses inferred with LSU and *cox1* showed unequivocally that Monorchiidae is monophyletic, coinciding with recent phylogenetic studies, adding to a more robust classification scheme of the family [28, 30]. Our study reports for the first-time DNA sequences for species of genus *Alloinfundiburictus*. In the phylogeny inferred with LSU, *A. haemuli* was the sister taxon of *Monorchis monorchis*, although with moderate to low nodal support, and with a long branch as reported by Wee *et al*. [28, 30]. Given that no *cox1* sequence is available for *M. monorchis* to corroborate the obtained LSU topology, in the *cox1* tree *A. haemuli* was yielded as the sister taxa of *Lobucirruatus infloresco*. Additional molecular data are still needed for the remaining 10 species of *Alloinfundiburictus* which parasitise fishes of families Haemulidae, Lethrinidae, Labridae and Serranidae distributed worldwide, in order to resolve the phylogeny and validate the monophyletic nature of the genus [28].

Only two species of *Sinistroporomonorchis* were sequenced for LSU before this study, *S.* *glebulentus* and *S. lizae*. This genus comprises two major clades, with a wide spectrum of genetic interspecific variation. The largest genetic divergence within *Sinistroporomonorchis* species was found between *S. lizae*, and *S. yucatanensis* n. sp. with *S. minutus* n. sp., with 6.3–7%. This divergence is very high in comparison with that of other species of monorchiid genera such as *Genolopa*. Divergence of LSU sequences among species of this genus range from 1.6–4.9% [18]. Morphologically, characters of the newly described species are consistent with the description of *Sinistroporomonorchis*, as mentioned in the remarks section. In addition, ecological characters also supported this assignation, since the two previously recognised species of *Sinistroporomonorchis* were also described from mugilids. The only lineage within *Sinistroporomonorchis* that does not parasitise a mugilid is *Sinistroporomonorchis* sp. (GenBank MN984477), reported as a parasite of the atherinopsid *Menidia menidia* off the coast of New Jersey, USA [18]. Unfortunately, these authors pointed out that their specimens were in poor condition for species-level identification and assigned them as “*Lasiotocus”* sp. However, our study showed that the species nested within *Sinistroporomonorchis*. A detailed analysis including more specimens is still needed to corroborate the morphological traits of this lineage and determine if it also represents a new species.

Our study contributes to the knowledge of the biodiversity of monorchiids infecting marine fishes. Clearly, more species remain to be described since they are found in different fish families such as Carangidae, Chaetodontidae, Mullidae, Sparidae, Serranidae, Ophichthidae, Haemulidae and at least another 30 fish families [32]. Sequencing work is also needed to expand the molecular library of this group of digeneans to aid in a robust taxonomic classification.

## Supplementary material

The supplementary material for this article can be found at https://www.parasite-journal.org/10.1051/parasite/2023015/olm. *Table S1*. Sequences from GenBank used for the phylogenetic analysis. *Table S2*. Pairwise nucleotide sequence comparisons between taxa for the aligned LSU rDNA sequences (1292 nt) (below the diagonal) and for *cox1* sequences (588 nt). The genetic intraspecific divergence is shown in bold.

## Financial Support

This research was supported by grants from the Programa de Apoyo a Proyectos de Investigación e Inovación Tecnológica (PAPIIT-UNAM IN212621), and the Consejo Nacional de Ciencia y Tecnología (CONACYT A1-S-21694) to GPPL.

## Conflict of interest

Authors declare no conflict of interest.
