# Supplementary material for: Monorchiids (Digenea, Trematoda) of fishes in the Yucatán Peninsula, Mexico, with the description of three new species based on morphological and molecular data
Source: Parasite. 2023 May 14;30:15. doi: 10.1051/parasite/2023015 (PMC10184649; doi:10.1051/parasite/2023015)
Supplement: Supplementary file 1 — Table S1. Sequences from GenBank used for the phylogenetic analysis. [file parasite-30-15-s1.pdf]

Table S1. Sequences from GenBank used for phylogenetic analysis in the present study.

| Family | Species | Host | GenBank accesion numbres | | Reference (s) |
|---|---|---|---|---|---|
| **Out Group** | | | **LSU** | ***cox1*** | |
| **Haploporidae** | *Saccocoelioides lamothei* Aguirre-Macedo & Violante-González, 2008 | *Poecilia gillii* | | MW283228 | González-García et al. 2021 |
| **Deropristidae** | *Skrjabinopsolus nudidorsalis* Sokolov, Voropaeva & Atopkin, 2020 | *Acipenser ruthenus* | MN700996 | | Sokolov et al . 2020 |
| **Lissorchiidae** | *Asaccotrema vietnamiense* Sokolov & Gordeev, 2019 | *Rasbora paviana* | MK863409 | | Sokolov and Gordeev, 2019 |
| | *Asymphylodora perccotti* Besprozvannykh, Ermolenko & Atopkin, 2012 | *Perccottus glenii* | FR822715 | | Besprozvannykh et al. 2012 |
| | *Asymphylodora progenetica* Serkova & Bykhowskii, 1940 | *Carpiodes cyprinus* | MT103400 | | Petkeviciute et al. 2020 |
| | *Palaeorchis incognitus* Szidat, 1943 | | MT103408 | | Petkeviciute et al. 2020 |
| | *Lissorchis kritskyi* Barnhart & Powell, 1979 | *Rutilus rutilus* | AY222250 | | Olson et al. 2003 |
| **Monorchiidae** | | | | | |
| Anamonorchiinae Yamaguti, 1970 | *Cableia pudica* Bray, Cribb & Barker, 1996 | *Cantherhines pardalis* | AY222251 | | Olson et al. 2003 |
| Hurleytrematinae Yamaguti, 1958 | *Helicometroides gabrieli* Wee, Cribb, Shirakash & Cutmore, 2022 | *Plectorhinchus chrysotaenia* | OM230129 | OM156428 | Wee et al. 2022 |
| | *Helicometroides longicollis* Yamaguti, 1934 Lineage 1 | *Diagramma pictum labiosum* | KJ658287 | OM156435 | Searle et al. 2014 |
| | *Helicometroides longicollis* Yamaguti, 1934 Lineage 2 | *D. pictum pictum* | OM230134 | OM156437 | Searle et al. 2014 |
| | *Helicometroides murakamii* Wee, Cribb, Shirakash & Cutmore, 2022 | *D. pictum pictum* | OM230128 | OM156426 | Searle et al. 2014 |

| | | | | |
|---|---|---|---|---|
| *Helicometroides wardae* Wee, Cribb, Shirakash & Cutmore, 2022 | *Plectorhinchus flavomaculatus* | OM230130 | OM156429 | Searle et al. 2014 |
| *Hurleytrematoides bartolii* Nahhas & Grewal, 1999 | *Heniochus sing* | | JN969442 | McNamara et al. 2014 |
| *Hurleytrematoides boucheti* McNamara, Adlard, Bray, Sasal & Cribb, 2012 | *Heniochus chrysostomus* | | JN969444 | McNamara et al. 2014 |
| *Hurleytrematoides combesi* McNamara, Adlard, Bray, Sasal & Cribb, 2012 | *Chaetodon unimaculatus* | | JN969446 | McNamara et al. 2014 |
| *Hurleytrematoides coronatus* Manter & Pritchard, 1961 | *Chaetodon vagabundus* | | JN969457 | McNamara et al. 2014 |
| *Hurleytrematoides chaetodoni* (Manter, 1942) Yamaguti, 1954 | *Chaetodon striatus* | MH244116 | | Andres et al. 2018 |
| *Hurleytrematoides deblocki* McNamara, Adlard, Bray, Sasal & Cribb, 2012 | *Forcipiger flavissimus* | | JN969462 | McNamara et al. 2014 |
| *Hurleytrematoides faliexae* McNamara & Cribb, 2011 | *Chaetodon ephippium* | | JN969469 | McNamara et al. 2014 |
| *Hurleytrematoides fijiensis* Nahhas & Grewal, 1999 | *Chaetodon citrinellus* | | JN969474 | McNamara et al. 2014 |
| *Hurleytrematoides galzini* McNamara & Cribb, 2011 | *Forcipiger flavissimus* | MK501988 | | Wee et al. 2019 |
| *Hurleytrematoides justinei* McNamara & Cribb, 2009 | *Canthigaster valentini* | | JN969476 | McNamara et al. 2014 |
| *Hurleytrematoides kulbickii* McNamara, Adlard, Bray, Sasal & Cribb, 2012 | *Chaetodon citrinellus* | | JN969478 | McNamara et al. 2014 |
| *Hurleytrematoides loi* McNamara & Cribb, 2011 | *Chelmon rostratus* | MK501989 | JN969481 | Wee et al. 2019; McNamara et al. 2014 |
| *Hurleytrematoides* | *Chaetodon* | MZ323087 | MZ318261 | Wee et al. 2021 |

| | | | | | |
|---|---|---|---|---|---|
| | *morandi* McNamara & Cribb, 2011 | *lunula* | | | |
| | | *Chaetodon vagabundus* | | JN969498 | McNamara et al. 2014 |
| | *Hurleytrematoides pasteuri* McNamara, Adlard, Bray, Sasal & Cribb, 2012 | *Heniochus acuminatus* | | JN969500 | McNamara et al. 2014 |
| | *Hurleytrematoides prevoti* Nahhas & Grewal, 1999 | *Chaetodon lineolatu* | | JN969501 | McNamara et al. 2014 |
| | *Hurleytrematoides sasali McNamara & Cribb, 2011* | *Coradion altivelis* | | JN969505 | McNamara et al. 2014 |
| | *Hurleytrematoides zebrasomae* Nahhas & Grewal, 1999 | *Heniochus chrysostomus* | | JN969507 | McNamara et al. 2014 |
| | *Hurleytrematoides* sp. | *Chaetodon melannotus* | | JN969509 | McNamara et al. 2014 |
| | *Provitellus chaometra* Wee, Cutmore & Cribb, 2019 | *Gnathanodon speciosus* | MK501984 | | Wee et al. 2019 |
| | *Provitellus infibrova* Wee, Cutmore & Cribb, 2019 | *Gnathanodon speciosus* | MK501986 | | Wee et al. 2019 |
| | *Provitellus infrequens* Wee, Cutmore & Cribb, 2019 | *Gnathanodon speciosus* | MK501985 | | Wee et al. 2019 |
| | *Provitellus turrum* Dove & Cribb, 1998 | *Pseudocaranx dentex* | AY222253 | | Olson et al. 2003 |
| | *Pseudohurleytrema yolandae* Wee, Crouch, Cutmore & Cribb, 2020 | *Tripodichthys angustifrons* | MT649300 | MT648485-86 | Wee et al. 2020 |
| Monorchiinae Odhner, 1911 | *Allobacciger annulatus* Wee, Cutmore, Sasal & Cribb, 2020 | *Centropyge tibicen* | MK955782 | MK975248-49 | Wee et al. 2020 |
| | *Allobacciger brevicirrus* Wee, Cutmore, Sasal & Cribb, 2020 | *Scolopsis bilineata* | MK955781 | MK975247 | Wee et al. 2020 |
| | *Allobacciger centropygis* Machida & Uchida, 2001 | *Centropyge flavissimus* | | JN969510 | McNamara et al. 2014 |
| | *Allobacciger polynesiensis* Wee, Cutmore, Sasal & Cribb, 2020 | *Centropyge flavissima* | MK955780 | MK975245 | Wee et al. 2020 |
| | *Ancylocoelium typicum* Nicoll, 1912 | | AY222254 | | Olson et al. |

| | | | | |
|---|---|---|---|---|
| | | | | 2003 |
| *Diplomonorchis leiostomi* Hopkins, 1941 | *Leiostomus xanthurus* | AY222252 | | Olson et al. 2003 |
| *Genolopa ampullacea* Linton, 1910 | *Haemulon macrostomum* | MN984474 | | Panyi et al. 2020 |
| *Genolopa minuscula* Panyi, Curran & Overstreet, 2020 | *Anisotremus surinamensis* | MN984472 | | Panyi et al. 2020 |
| *Genolopa vesca* Panyi, Curran & Overstreet, 2020 | *Haemulon sciurus* | MN984471 | | Panyi et al. 2020 |
| *Gerricola queenslandensis* Wee, Cutmore & Cribb, 2021 | *Gerres oyena* | MZ271999 | MZ295281 | Wee et al. 2021 |
| | *Gerres subfasciatus* | | MZ295277 | |
| *Infundiburictus arrhichostoma* (Searle, Cutmore & Cribb, 2014) Wee, Cutmore, Pérez-del-Olmo & Cribb, 2020 | *D. pictum labiosum* | KJ658289 | | Searle et al.2014 |
| *Lasiotocus mulli* (Stossich, 1883) Odhner, 1911 | *Mullus surmuletus* | MT669011 | MT665981 | Wee et al. 2020 |
| *Lasiotocus trachinoti* Overstreet & Brown, 1970 | *Trachinotus carolinus* | MN984478 | | Panyi et al. 2020 |
| | *Donax fossor* (Cercariae) | | MW628338 | Hill-Spanik et al. 2021 |
| *'Lasiotocus sp'* | *Menidia menidia* | MN984477 | | Panyi et al. 2020 |
| *Lasiotocus choanura* (Hopkins, 1958) Hill-Spanik, Sams, Connors, Bricker & de Buron, 2021 | *Donax cf. fossor* | | MN389451-52 | Hill-Spanik et al. 2021 |
| *Lobucirruatus infloresco* Wee, Cribb & Cutmore, 2022 | *Parupeneus spilurus* | OM891782 | OM868215 | Wee et al. 2022 |
| *Madhavia fellaminuta* Wee, Cutmore & Cribb, 2018 | *Upeneus tragula* | MG920219 | | Wee et al. 2018 |

| | | | | | |
|---|---|---|---|---|---|
| | *Monorchis lewisi* Cribb, Wee, Bray & Cutmore, 2018 | *Acanthopagrus australis* | MF503309 | | Cribb et al. 2018 |
| | *Monorchis monorchis* (Stossich, 1890) Looss, 1902 | *Diplodus vulgaris* | AF184257 | | Tkach et al. 2001 |
| | *Ovipusillus geminus* Wee, Cutmore & Cribb, 2019 | *Gnathanodon speciosus* | MF501987 | | Wee et al. 2019 |
| | *Ovipusillus mayu* Dove & Cribb, 1998 | *Gnathanodon speciosus* | MF503310 | | Cribb et al. 2018 |
| | *Paralasiotocus abstrusus* Wee, Cribb & Cutmore, 2022 | *Plectorhinchus albovittatus* | OM891779 | OM868212 | Wee et al. 2022 |
| | *Paralasiotocus tectus* Wee, Cribb & Cutmore, 2022 | *Plectorhinchus albovittatus* | OM891778 | OM868214 | Wee et al. 2022 |
| | *Postmonorchis orthopristis* Hopkins, 1941 | *Haemulon flavolineatum* | MN984475 | | Panyi et al. 2020 |
| | *Proctotrema addisoni* Searle, Cutmore & Cribb, 2014 | *D. pictum labiosum* | KJ658291 | | Searle et al. 2014 |
| | *Proctotrema prominens* Wee, Cribb y Cutmore, 2022 | *Plectorhinchus albovittatus* | OM891777 | OM868216 | Wee et al. 2022 |
| | *Retroporomonorchis pansho* Wee, Cribb, Cutmore & Martin, 2020 | *Lutjanus fulvus* | MT672340 | MT671498 | Wee et al. 2020 |
| | *Sinistroporomonorchis glebulentus* (Overstreet, 1971) Wee, Cutmore, Pérez-del-Olmo & Cribb, 2020 | *Mugil curema* | MN984476 | | Panyi et al. 2020 |
| | *Sinistroporomonorchis lizae* (Liu, 2002) Wee, Cutmore, Pérez-del-Olmo & Cribb, 2020 | *Moolgarda perusii* | LN831720 | | Atopkin et al. 2017 |
| **Unknown** | *Parachrisomon delicatus* (Manter & Pritchard, 1961) Madhavi, 2008 | *Upeneus tragula* | MG920218 | | Wee et al. 2017 |
| | *Monorchiidae* sp. | *Jactellina clathrata* | MZ272001 | MZ295282 | Wee et al. 2020 |
