# Supplementary material for: Monorchiids (Digenea, Trematoda) of fishes in the Yucatán Peninsula, Mexico, with the description of three new species based on morphological and molecular data
Source: Parasite. 2023 May 14;30:15. doi: 10.1051/parasite/2023015 (PMC10184649; doi:10.1051/parasite/2023015)
Supplement: Supplementary file 2 — Table S2. Pairwise nucleotide sequence comparisons between taxa for the aligned LSU rDNA sequences (1292 nt) (below the diagonal) and for cox1 sequences (588 nt). The genetic intraspecific divergence is shown in bold. [file parasite-30-15-s2.pdf]

Table S2. Pairwise nucleotide sequence comparisons between taxa for the aligned LSU rDNA sequences (1292 nt) (below the diagonal) and for *cox1* sequences (588 nt). In bold is represented the genetic intraspecific divergence.

LSU	1 *Lobucirruatus infloresco*	2 *Allobacciger* spp.	3 *Alloinfundiburictus haemuli*	4 *Monorchis monorchis*	5 *Sinistroporomonorchis* sp. (‘*Lasiotocus* sp.’)	6 *S. lizae*	7 *S. glebulentus*	8 *S. mexicanus* n. sp.	9 *S. minutus* n. sp.	10 *S. yucatanensis* n. sp.
1 *Lobucirruatus infloresco*	–	–	–	–	–	–	–	–	–	
2 *Allobacciger* spp.	8.3-8.8	**1.5-2.3**	–	–	–	–	–	–	–	
3 *Alloinfundiburictus haemuli*	11.3-13.6	11.7-15.4	**0-0.3**	–	–	–	–	–	–	
4 *Monorchis monorchis*	11.1	12.3-13.3	12.8-17	**–**	–	–	–	–	–	
5 *Sinistroporomonorchis* sp. (‘*Lasiotocus* sp.’)	8.6	10.6-11.4	11.1-14.2	10	**–**	–	–	–	–	
6 *S. lizae*	9.3-9.7	11.2-12.7	12.1-16.2	10.6-11.2	4.2-5	**–**	–	–	–	
7 *S. glebulentus*	9.3-9.7	11.2-12.7	12.1-16.2	10.6-11.2	4.2-5	2.1-2.2	**0**	–	–	
8 *S. mexicanus* n. sp.	9.3-9.7	11.2-12.7	12.1-16.2	10.6-11.2	4.2-5	2.7-2.8	0.9-1	**0**	–	
9 *S. minutus* n. sp.	9-9.5	10-11.6	10.4-14.5	10.9-11.9	5.2-5.7	6.3-7	5.7-6.4	6-6.9	**0-0.1**	
10 *S. yucatanensis* n. sp.	9-9.5	10-11.6	10.4-14.5	10.9-11.9	5.2-5.7	6.3-7	5.7-6.4	6-6.9	2.6-2.9	**0-0.08**

cox 1	1 *Lobucirruatus infloresco*	2 *Allobacciger* spp.	3 *Alloinfundiburictus haemuli*	7 *S. glebulentus*	8 *S. mexicanus* n. sp.	9 *S. minutus* n. sp.	10 *S. yucatanensis* n. sp.
1 *Lobucirruatus infloresco*		–	–	–	–	–	
2 *Allobacciger* spp.	18.3-20.4	**15.1**	–	–	–	–	
3 *Alloinfundiburictus haemuli*		17.8-20.4	**0.3**	–	–	–	
7 *S. glebulentus*	17.3-18.1	18-22.1	17.2-18.3	**0.2-0.8**	–	–	
8 *S. mexicanus* n. sp.	17.3-18.1	18-22.1	17.2-18.3	9-9.4	**0**	–	
9 *S. minutus* n. sp.	19.1-22.5	18-24.4	19.2-21.4	14.9-16.4	14.4-18.2	**9.2**	
10. *S. yucatanensis* n. sp.	19.1-22.5	18-24.4	19.2-21.4	14.9-16.4	14.4-18.2	14.4-15.6	**0.1-1.1**
